# Phenotype and function of IgE-binding monocytes in equine *Culicoides* hypersensitivity

**DOI:** 10.1371/journal.pone.0233537

**Published:** 2020-05-22

**Authors:** Elisabeth M. Larson, Susanna Babasyan, Bettina Wagner

**Affiliations:** Department of Population Medicine and Diagnostic Sciences, College of Veterinary Medicine, Ithaca, New York, United States of America; Massey University, NEW ZEALAND

## Abstract

Human IgE-binding monocytes are identified as allergic disease mediators, but it is unknown whether IgE-binding monocytes promote or prevent an allergic response. We identified IgE-binding monocytes in equine peripheral blood as IgE+/MHCII^high^/CD14^low^ cells that bind IgE through an FcεRI αɣ variant. IgE-binding monocytes were analyzed monthly in *Culicoides* hypersensitive horses and nonallergic horses living together with natural exposure to *Culicoides* midges. The phenotype and frequency of IgE-binding monocytes remained consistent in all horses regardless of *Culicoides* exposure. All horses upregulated IgE-binding monocyte CD16 expression following initial *Culicoides* exposure. Serum total IgE concentration and monocyte surface IgE densities were positively correlated in all horses. We also demonstrated that IgE-binding monocytes produce IL-10, but not IL-4, IL-17A, or IFN-γ, following IgE crosslinking. In conclusion, we have characterized horse IgE-binding monocytes for the first time and further studies of these cells may provide important connections between regulation and cellular mechanisms of IgE-mediated diseases.

## Introduction

The most prevalent, naturally occurring allergy in horses is known as *Culicoides (Cul)* hypersensitivity. This disease is also frequently called insect bite hypersensitivity (IBH), summer eczema, summer seasonal recurrent dermatitis, or sweet itch [1–9]. Allergic horses suffer from pruritus, dermatitis and hair loss in response to salivary proteins of *Cul* midges [7,10]. Reactions range in severity and can be debilitating for the horse. This hypersensitivity reaction is mediated by the production of IgE and subsequent sensitization of mast cells and basophils by binding of IgE to the high-affinity IgE receptor (FcεRI) on the surface of these cells [10–16]. Exposure to allergen induces crosslinking of allergen-specific IgE/FcεRI complexes on mast cells, resulting in rapid degranulation and an immediate inflammatory response [17,18].

A variant of FcεRI is expressed on human antigen-presenting cells including monocytes [19,20]. The trimeric FcεRI on these cells contains α and γ receptor chains only (αγ_2_) and is lacking the β chain, which is part of the tetrameric receptor (αβγ_2_) on basophils and mast cells [15–18,21–24]. The β chain is a transmembrane protein that acts as a signal amplifier of the receptor. Alternative splice variants of this receptor modulate mast cell function in humans and exacerbate disease [25,26]. In humans, αγ_2_ FcεRI is involved in antigen recognition [23,27] where allergen is internalized via receptor-bound IgE/allergen complexes, intracellularly processed, and ultimately presented via major histocompatibility complex class II (MHCII) molecules to T cells in the draining lymph node [20,28–30].

Monocytes exhibit highly plastic functions and are responsible for rapid migration towards sites of inflammation, phagocytosis of invading pathogens or allergens, and presentation of foreign epitopes to T cells in regional lymph nodes [31,32]. Importantly, monocytes secrete cytokines and chemokines during these processes that educate the immune response to appropriately respond to invading pathogens or allergens [33]. While *Cul* allergens are inherently innocuous [34–36], the regulatory response fails to induce adequate tolerance to allergens in horses with *Cul* hypersensitivity [37]. The exact mechanisms of tolerance failure in allergic individuals still remain unknown. Nevertheless, the establishment of successful treatment strategies of clinical allergy in horses will likely depend on further understanding of their immune mechanisms leading to development of allergen tolerance.

In humans, IgE-binding monocytes are present in all individuals but express higher densities of FcεRI in atopic dermatitis patients, compared with healthy controls [19,20,38]. Conflicting evidence supports either an inflammatory [39,40] or anti-inflammatory [40,41] role for this subpopulation of IgE-binding monocytes. Here, we present evidence of a similar subpopulation of IgE-binding monocytes in equine peripheral blood mononuclear cells (PBMC). Specifically, this study focused on characterizing the phenotype and functional role of equine IgE-binding monocytes in horses with or without *Cul* hypersensitivity. We also developed a high purity sorting protocol of IgE-binding monocytes that enabled the characterization of intrinsic properties of these cells. We asked the following questions: (1) What surface and secreted proteins characterize IgE-binding monocytes in horses? (2) Do cell numbers and/or does cell surface protein expression change during seasonal environmental exposure to *Cul* in IgE-binding monocytes of allergic and nonallergic horses? (3) What cytokines are produced by IgE-binding monocytes in response to IgE-dependent stimulation?

## Materials and methods

### Animals, clinical allergy scoring, and blood sampling

Horses studied for this approach were either clinically healthy or were affected with *Culicoides* (*Cul*) hypersensitivity. Allergic (n = 7) and nonallergic (n = 7) groups both consisted of sex-matched Icelandic horses (6 mares and 1 gelding per group). The two geldings were 6-years and the mares were ages 6–15 years. Peripheral blood mononuclear cells (PBMC) from these horses were used for all flow cytometry experiments and RNA extractions. In addition, PBMC from allergic (n = 8) and nonallergic (n = 8) groups composed of one stallion (6 years), two geldings (7 and 8 years), and 13 mares (6–16 years) were used for cytokine secretion experiments. All horses lived together in the same geographic location and environment in Ithaca, NY for at least two years prior to this study. They were kept on large pastures 24/7 with free access to water, mineral salt blocks, grass in the summer and grass hay in the winter months. All horses were annually vaccinated against rabies, tetanus, West Nile virus, and Eastern and Western Encephalitis virus. They were also dewormed twice a year. Vaccination and deworming were performed for all horses on the same days.

Clinical scores [42] were assigned based on pruritus (0–3), alopecia (0–4), and skin irritation (0–3), and were given to each horse every 2–4 weeks for the duration of the study. All allergic horses displayed clinical signs of allergy (clinical scores ≥ 3) for at least one summer of *Cul* exposure before and also during the study. *Cul*-specific hypersensitivity was confirmed by intradermal skin testing with *Cul* whole body extract (WBE; Stallergenes Greer Inc., Cambridge, MA.) in comparison to saline and histamine controls as previously described [15,17]. Allergic horses developed an immediate skin reaction to *Cul* WBE. Nonallergic horses never exhibited clinical signs scoring above 3 (Table 1), and also did not react to intradermal *Cul* WBE injections. Blood samples were obtained from the V. jugularis using the BD Vacutainer system (Becton Dickinson, Franklin Lakes, NJ). Both heparinized blood samples and serum samples were collected once a month for one year (April 2018 to March 2019).

**Table 1:** Allergic horses (*Culicoides* hypersensitivity) exhibited clinical signs during *Cul* exposure while nonallergic horses living in the same environment did not show clinical signs of allergy.

		Nonallergic Horses (n = 7)	Allergic Horses (n = 7)
**Age (years), median (range)**		7 (5–12)	12 (7–15)
**Clinical score, median (range)**	**without *Culicoides* exposure** ^**a**^	0 (0)	0 (0)
	**with *Culicoides* exposure** ^**a**^	0.3 (0–2)	(3–8)

^a^*Cul* exposure months included June–September.

All animal procedures were carried out in accordance with the recommendation in the Guide for the Care and Use of Laboratory Animals of the National Institutes of Health. The animal protocol was approved by the Institutional Animal Care and Use Committee at Cornell University (protocol #2011–0011). The study also followed the Guide for Care and Use of Animals in Agricultural Research and Teaching.

### Cell isolation and fixation

PBMC were isolated from heparinized blood of all horses by density gradient centrifugation of the cell-rich plasma (Ficoll-PaqueTM Plus, GE Healthcare, Piscataway, NJ). For flow cytometry analysis, heparinized blood was allowed to settle at room temperature for 1–2 hours before collection of cell-rich plasma. For cell sorting, heparinized blood sat at room temperature for 2 hours to allow for maximal separation of neutrophils and erythrocytes from the other cells in the cell-rich plasma. Following density gradient isolation, PBMC were washed in PBS and either used immediately for cell sorting or fixed in 2% (v/v) formaldehyde (SigmaAldrich) for 20 minutes at room temperature prior to cell surface staining and flow cytometry. PBMC for RNA isolation were isolated as described above, pelleted, and snap frozen in liquid nitrogen.

### Cell surface staining and flow cytometry analysis

Fixed PBMC were incubated for 15 minutes at room temperature with multiple antibody master mixes (Table 2) in PBS-BSA (0.5% (w/v) BSA, 0.02% (w/v) NaN3) and subsequently incubated with fluorochrome conjugated or biotinylated monoclonal antibodies (mAbs). All mAbs were developed in mice and isotyped as mouse IgG1, unless otherwise specified. Antibodies were conjugated to Alexa fluorochrome 647, Alexa fluorochrome 488, phycoerythrin (PE) or biotin according to the manufacturer’s protocols (ThermoFisher). Streptavidin-PerCP-cyanin 5.5 (Biolegend) and Streptavidin-PE (Jackson ImmunoResearch) were used to label biotinylated mAbs. Master mixes 1 and 2 were used for longitudinal analysis of IgE-binding monocyte surface protein expression, and master mix 3 was used for IgE receptor expression. Master mix 7 was used for measurement of IgE mAb median fluorescence intensity (MFI).

**Table 2:** Antibody master mixes used for flow cytometric analysis or fluorescence activated cell sorting of IgE-binding monocytes.

Master Mix	Alexa Fluor 647	Alexa Fluor 488	Phycoerythrin	PerCP-Cy5.5	Violet
1	Equine CD16^a^	Equine IgE^f^	Equine MHCII^g^	Equine CD14^e^^,^^i^	N/A
2	Human CD163^b^				
3	Equine CD23^c^				
4	CD16^a^ and CD163^b^				Live/Dead
5	Equine IL-10^d^		Equine CD14^e^^,^^h^	N/A	N/A
6	Goat-anti-mouse Fab_2_		Equine MHCII^g^^,^^h^		
7	Equine CD14^e^				

^a^Clone 9G5 [43]

^b^Clone Ber-Mac3 [44]

^c^Clone 51–3 [45]

^d^Clone 165–2 [46]

^e^Clone 105 [47]

^f^Clone 176 [13]

^g^Clone cz11 [48]

^h^mAb was biotinylated and fluorescently labeled with streptavidin-PE

^i^mAb was biotinylated and fluorescently labeled with streptavidin-PerCP-Cy5.5

For sample analysis, 100,000 events per sample were recorded on a FACS Canto II flow cytometer (BD Biosciences, San Diego, CA) and data analysis was performed with FlowJo version 10.4 (FlowJO LLC, Ashland, OR, USA). Compensation was calculated with input from single stained UltraComp beads (ThermoFisher). IgE-binding monocytes (also referred to as IgE+ monocytes or IgE+/MHCII^high^/CD14^low^) in the monocyte/large lymphocyte forward (FSC) and side scatter (SSC) were analyzed quantitatively. CD16 and CD163 expression of IgE+/MHCII^high^/CD14^low^ cells was also calculated. Median fluorescence intensity (MFI) of anti-IgE (AlexaFluor 488) was calculated on IgE-binding monocytes (IgE+/ CD14^low^).

### Magnetic and fluorescence activated cell sorting

Following PBMC isolation, CD14+ cells were enriched using magnetic cell sorting (MACS system, Miltenyi Biotech, Auburn, CA). 1x10^8^ cells were incubated with unconjugated 1.6 μg anti-equine CD14 mAb (clone 105) [47] in 1.2 ml PBS-BSA for 15 minutes on ice, followed by incubation with 35 μl rat anti-mouse IgG1-coated magnetic beads (Miltenyi Biotech, Auburn, CA) in 350 μl total volume. Cells were separated over an LD column. MACS sorting enriched total CD14+ monocytes from 8–52% (median 28.1%) in PBMC to 80–96% (median 92.6%) in the CD14+ fraction. Cells were used for subsequent fluorescence activated cell sorting (FACS). To determine sorting purity after MACS sorting, an aliquot of live cells was incubated in PBS-BSA with goat-anti-mouse Fab_2_ to label the mouse-anti-horse CD14 mAb (Table 2, master mix 6). These aliquots were then fixed in 2% (v/v) formaldehyde, washed, and further incubated in PBS-BSA with anti-equine IgE and MHCII mAbs as described above (Table 2, master mix 6). Sorting purity was determined by reading 100,000 events per sample on a FACS Canto II flow cytometer as described above.

For FACS following CD14 enrichment, the CD14+ fraction was further incubated with mAbs (Table 2, master mix 4), including a viability marker (ThermoFisher), to isolate IgE-binding monocytes. IgE mAb 176 is a monoclonal antibody that binds specifically to equine IgE [13], but does not induce crosslinking of the IgE receptor on basophils or mast cells [15–18]. We therefore used clone 176 for FACS of live cells due to the fact that the antibody binding does not activate the monocytes through the IgE receptor. All sorting was performed at 4ºC to minimize any non-specific activation of monocytes. Cells were sorted on a BD FACS Aria Fusion Sorter at Cornell University’s Biotechnology Resource Center (BRC) Flow Cytometry Core. Compensation was calculated with input from single stained UltraComp beads (ThermoFisher) for the mAbs, and single stained amine reactive beads (ThermoFisher) for the viability dye. Sorting was performed through an 85 μm nozzle at 100 psi in a sterile hood. Anti-CD16 and anti-CD163 mAbs were conjugated to the same fluorochrome and analyzed as a dump gate. Live IgE+/MHCII^high^/CD14^low^/CD16-/CD163- cells were collected into cell culture medium (DMEM supplemented with 1% (v/v) non-essential amino acids, 2 mM L-glutamine, 50 μM 2-mercaptoethanol, 50 μg/ml gentamicin, 100 U/ml penicillin, 100 μg/ml streptomycin (all from ThermoFisher Scientific, Waltham, MA, USA), and 10% FCS (Atlanta biological, Flowery Branch, GA, USA) at room temperature. Following sorting, IgE-binding monocytes were counted and tested for viability using trypan blue exclusion. All samples resulted in >95.5% viable (average 99.2% live cells).

### RT-PCR of FcεRI transcripts

Aliquots of 1x10^6^ PBMC and 1x10^3^-5x10^5^ sorted IgE-binding monocytes were collected in 1.5 ml Eppendorf tubes and pelleted for 5 minutes at 300 xg. Cell pellets were washed in PBS, dried by vacuum aspiration, snap frozen in liquid nitrogen, and stored at -80ºC. Cell pellets were then homogenized with a QIAshredder kit (QIAGEN) and RNA was extracted using a RNeasy Mini kit (QIAGEN) and DNase digestion (QIAGEN). cDNA was generated using random hexamers (Superscript First Strand Synthesis System, Invitrogen, CA) and gene specific primers were used to amplify unique regions of each equine FcεRI subunit gene with a Platinum^TM^ PCR SuperMix High Fidelity master mix (Invitrogen). The three equine genes and primer pairs were: FCERIA (FcεRIα; GenBank accession NM_001099767.2; bases 13–821, forward primer: AGGCCACAGAGGAGATGC, reverse primer: TGACATCAGTTCTTTCTCGGGT), FCERIB (FcεRIβ; GenBank accession AJ318332.1; bases 36–779; forward primer: CCAGAAAATAGGGCCAGAGC; reverse primer: ATGTTCTGGGCACAGCATTC), FCERIG (FcεRIγ; GenBank accession NM_001309482.1; bases 61–344; forward primer: CCAGCAGTGGTCTTGCTCTT; reverse primer: GGATGTGACCAAGGGCATCT). The FcεRI gamma chain (i.e. common Fc receptor gamma chain) is conserved in FcεRI and FcγRs, and therefore served as a positive control.

### *In vitro* stimulation and cytokine detection

CD14+ MACS enriched cells (6.5x10^5^ cells/well) were stimulated with 3 μg/ml anti-equine IgE clone 134 (IgE mAb 134) which induces crosslinking of the IgE receptor [15–18], 1:5 dilution of *Culicoides* whole body extract (WBE; Stallergenes Greer Inc., Cambridge, MA.), or 3 μg/ml anti-equine IgE clone 176 (IgE mAb 176) as a negative control which does not induce crosslinking [15]. CD14+ MACS enriched cells were also stimulated with 2.5 μg/ml pokeweed mitogen (PWM) (Sigma Aldrich, St. Louis, MO, USA) as a positive control, or culture media alone. Cells where incubated in 96-well flat-bottom plates (Corning Incorporated) at 37ºC, 5% CO_2_ for 24 and 48 hours before supernatants were collected and stored at 4ºC until analysis. Cytokine production was measured using a fluorescent bead-based multiplex to simultaneously detect equine IL-10, IL-4, IL-17, and IFN-ɣ. Assay was set up as previously described [33].

For visualization of IL-10 production by IgE-binding monocytes, CD14+ MACS enriched cells (1.8x10^6^ cells/dish) were plated in 35 mm MatTek No. 1.5 coverslip dishes (MatTek Corporation) and incubated in cell culture media or with 3 μg/ml IgE mAb 134 for 24 hours. A final concentration of 10 μg/ml Brefeldin A (Sigma Aldrich, St. Louis, MO, USA) was added during the last 4 hours of incubation before cells were fixed in MatTek wells in 2% (v/v) formaldehyde (SigmaAldrich). Following fixation, cells were incubated with mAbs described above in Table 2 (master mix 5). Cell images were obtained at Cornell University’s BRC facilities using a Zeiss LSM710 confocal microscope at 65x magnification and 16-bit image.

### Fluorescent bead assay for serum total IgE quantification

Peripheral blood was collected from all horses monthly into serum vacutainer tubes as described above. Tubes were left at room temperature for approximately 2–4 hours, then centrifuged at 2000 rpm for 5 minutes to allow serum separation. Serum was collected and stored at -20ºC until analyzed for total IgE levels.

Equine IgE mAb 176 [13] was coupled to fluorescent bead 35 (Luminex Corp., http://www.luminexcorp.com) following the recommended manufacturer’s protocol and as previously described [33]. The total IgE assay was designed similar to previous protocols [33]. Briefly, IgE mAb 176 coupled beads were vortexed and sonicated for 20 seconds and diluted in blocking buffer (PBS supplemented with 1% (w/v) BSA and 0.05% (w/v) sodium azide) to a final concentration of 5x10^4^ beads/ml. Native equine IgE purified from supernatant of equine B cell/murine X63-Ag8.653 myeloma cell hetero-hybridoma clone 37–1 was used as standard for quantification [49]. Concentration standard was prepared by diluting IgE 37–1 in blocking buffer using 5-fold dilutions with a concentration range from 3.2–10,000 ng/ml.

Millipore Multiscreen HTS plates (Millipore, Danvers, MA) were soaked with PBS-T (PBS supplemented with 0.1% (w/v) BSA, 0.02% (v/v) Tween 20 and 0.05% (w/v) sodium azide) using an ELx50 plate washer (Biotek Instruments Inc., Winooski, VT) for 3 minutes. PBS-T was aspirated from wells and replaced with 50 μl serum samples diluted 1:50 in blocking buffer or 50 μl of each diluted concentration standard. 50 μl of diluted IgE mAb 176 coupled #35 bead solution was added to each well and incubated on a shaker at room temperature in the dark for 30 minutes, and then washed with PBS-T. Biotinylated IgE mAb 134 was diluted to 8 μg/ml in blocking buffer and 50 μl was added to each well, incubated for 30 minutes as above, and washed with PBS-T. Streptavidin-phycoerythrin (Invitrogen, Carlsbad, CA) was diluted to 10 μg/ml in blocking buffer, 50 μl was added to each well, incubated for 30 minutes as above, and washed with PBS-T. Beads were resuspended with 100 μl/well blocking buffer and analyzed in a Luminex 200 instrument (Luminex Corp., http://www.luminexcorp.com). The data were reported as median fluorescent intensities (MFI) and total IgE concentration was calculated with a standard curve fit and logistic 5p formula (y = a+b/(1+(x/c)^d^)^f^) (Luminex 100 Integrated System 2.3).

### Statistical analysis

Data sets were tested by D’Agnostico and Pearson tests and determined to be mostly non-Gaussian distributed. Occasionally, one sample group was normally distributed and the other was not, in which case both groups were analyzed using non-Gaussian statistical tests. To compare clinical signs and percentages of IgE-binding monocytes between allergic and nonallergic groups, a Sidak multiple comparisons test was used. To compare IgE-binding monocytes between different timepoints, a Dunnett’s multiple comparisons test was used. A nonparametric Spearman rank correlation, and simple linear regression was calculated for all horses to compare IgE MFI on IgE binding monocytes and serum total IgE concentration. IL-10 production by allergic and nonallergic groups were medium corrected and compared by a Mann-Whitney test. P values <0.05 were considered significant. All graphs plot median and range unless specified otherwise. Analysis was performed with GraphPad Prism software version 8 (GraphPad Software Inc., La Jolla, CA, USA).

## Results

### Characterization of IgE-binding monocytes in the peripheral blood of allergic and nonallergic horses

**Fig 1:**
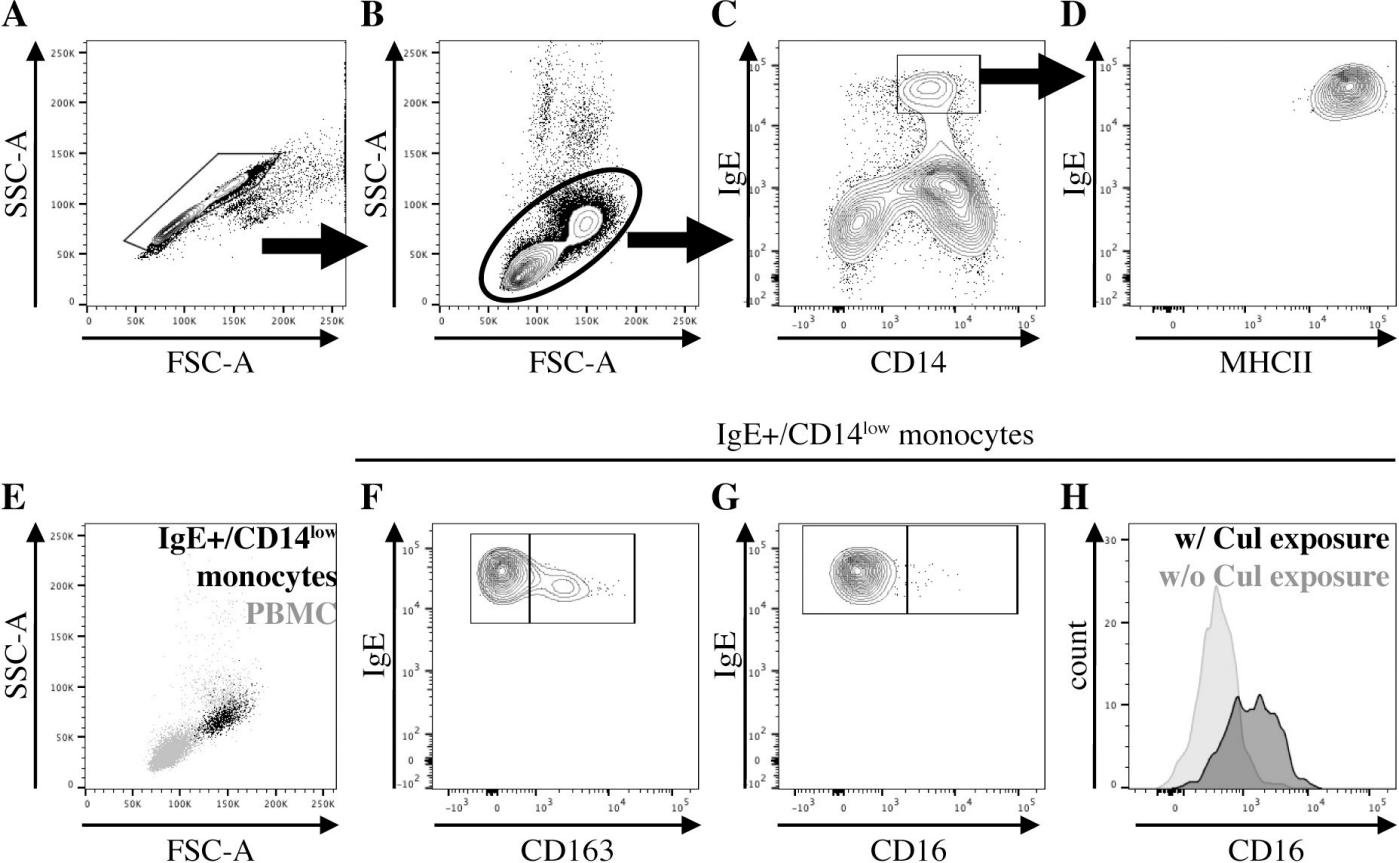
IgE-binding monocytes are IgE+/CD14^low^/MHCII^high^/CD16-/CD163-. PBMC were stained for different cell surface markers and IgE-binding monocyte surface protein expression was determined. (A) Doublet exclusion; (B) PBMC morphology gate; (C) CD14 and IgE expression on cells in the PBMC morphology gate; (D) MHCII expression on IgE+/CD14^low^ cells; (E) Forward (FSC) and side scatter (SSC) characteristics of IgE+/CD14^low^ cells (black) compared to PBMC (gray); (F) CD163 and (G) CD16 expression on IgE+/CD14^low^ cells; (H) Comparison of CD16 expression on IgE+/CD14^low^ cells during environmental *Cul* exposure and clinical signs of allergy in July (black), and while *Cul* were not in the environment and clinical allergy was resolved in March (gray).

Phenotyping of monocytes was performed by multi-color cell surface staining and flow cytometric analysis of PBMC from 14 adult horses (Table 1). All allergic (n = 7) and nonallergic horses (n = 7) expressed a similar population of IgE+ monocytes in peripheral blood throughout the year. Equine IgE+ monocytes were characterized by surface protein expression as IgE+/CD14^low^/MHCII^high^/CD16-/CD163- cells (Fig 1). A gating strategy to identify IgE+ monocytes was established on doublet excluded cells (Fig 1A). First, we gated for total PBMC by forward (FSC) and side scatter (SSC) (Fig 1B). IgE+ monocytes expressed CD14 with an overall slightly lower density than CD14+/IgE- monocytes. The IgE+ monocyte population was therefore called CD14^low^ (Fig 1C). IgE+ monocytes were further characterized as MHCII^high^ cells (Fig 1D) and showed high amounts of IgE on their surface (Fig 1C and 1D). These IgE+/CD14^low^ monocytes had high size and complexity by FSC/SSC (Fig 1E). The majority of IgE+ monocytes lacked expression of CD163 (Fig 1F) and CD16 (Fig 1G) while *Cul* was absent from the environment of the horses. During environmental *Cul* exposure, IgE+ monocytes in all horses began to express CD16 (Fig 1H).

**Fig 2:**
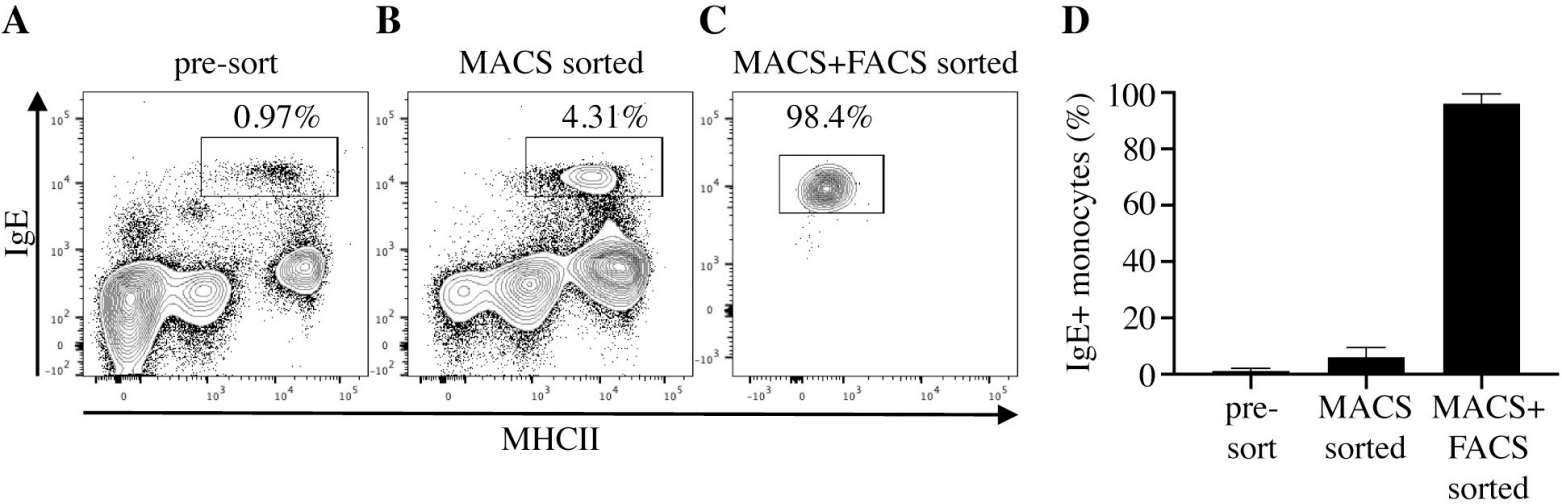
Magnetic and FACS sorting of IgE-binding monocytes from peripheral blood. IgE-binding monocytes were first enriched by magnetic cell sorting and subsequently purified by fluorescence activated cell sorting (FACS). Sorting purities were quantified by flow cytometry. (A) Unsorted cells; (B) CD14+ fraction after enrichment by magnetic cell sorting; (C) FACS sorted IgE-binding monocytes (IgE+/CD14^low^/MHCII^high^/CD16-/CD163-); (D) IgE-binding monocyte (IgE+/CD14^low^/MHCII^high^) purities after each sorting step. The graph shows medians and ranges of purities for sorted cells of 18 horses.

**Fig 3:**
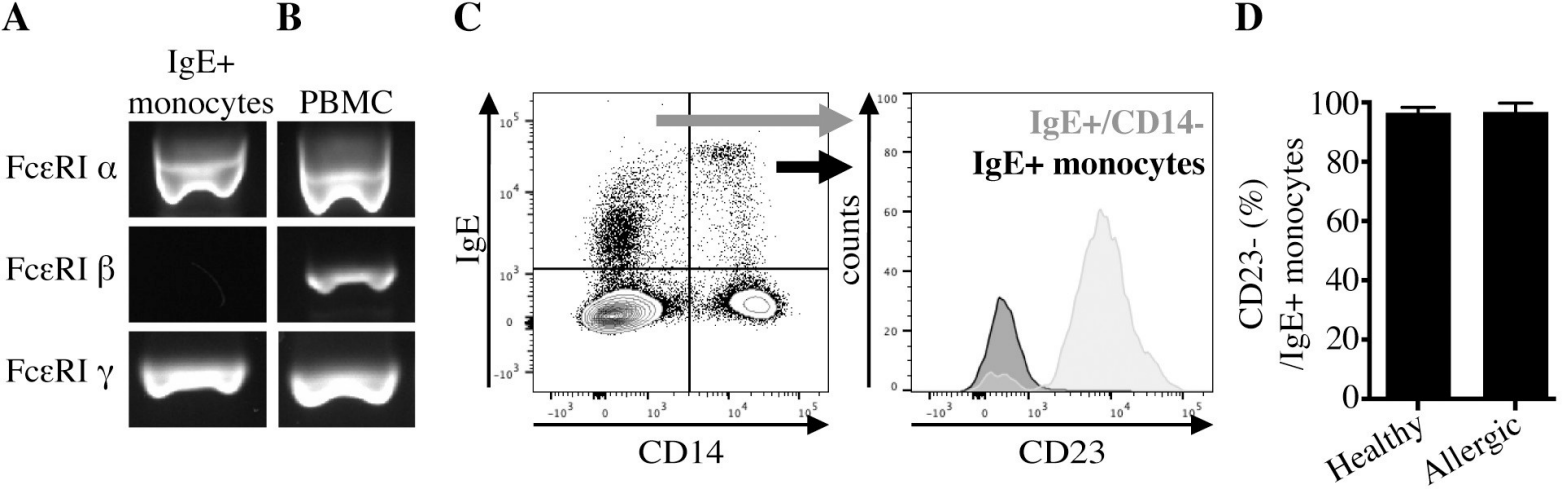
IgE-binding monocytes express αγ FcεRI and lack expression of the FcεRI β chain and CD23. FcεRI α, β, and γ subunit genes were amplified by PCR using gene specific primers. mRNA was extracted, and cDNA was transcribed and amplified from (A) purified IgE-binding monocytes (IgE+/CD14^low^/MHCII^high^/CD16-/CD163-); or (B) PBMC. PCR products from one representative horse are shown. The PCR was repeated with cells from two allergic and two nonallergic horses. (C) Cell surface staining of PBMC with an anti-CD23 mAb was performed and analyzed by flow cytometry. Cells were gated first by CD14 and IgE expression. FcεRII (CD23) expression was compared on gated IgE+/CD14- cells (gray) and IgE+ monocytes (black); (D) Percentages of CD23- IgE-binding monocytes by flow cytometry in allergic (n = 7) and nonallergic (n = 7) horses.

Expression of the different high-affinity IgE receptor (FcεRI) subunits was analyzed in monocytes to identify if IgE on equine monocytes is bound via FcεRI. Viable IgE+ monocytes were purified (median 95.6%; range 88.5–98.8% of 72 sorting procedures) using magnetic and subsequent fluorescence activated cell sorting (Fig 2). Sort purities of IgE+ monocytes were similar between allergic (median 95.6%, range 91.3–98.3%) and nonallergic (median 96.5%, range 92.8–99.5%) horse groups. mRNA from purified IgE+ monocytes and PBMC was isolated and FcεRI subunit cDNA was amplified using gene specific primers. IgE-binding monocytes in both allergic and nonallergic horses expressed mRNA transcripts for the α and γ subunits of the FcεRI receptor, but not for the β chain (Fig 3A). In contrast, PBMC, which included both IgE+ monocytes and basophils, expressed mRNA for all three receptor subunits α, β, and γ (Fig 3B). PCR products were submitted for sanger-sequencing and all sequences aligned to the corresponding equine FcεRI subunit genes (FCERIA, FCERIB, FCERIG).

In addition, low affinity IgE receptor FcεRII, also named CD23, expression on IgE+ monocytes was determined via cell surface staining and flow cytometric analysis of unsorted PBMC. While IgE+/CD14- cells were FcεRII/CD23+, IgE+ monocytes (IgE+/CD14^low^, see Fig 1A–1D for gating strategy) were FcεRII/CD23- (Fig 3C). IgE+/CD14-/CD23+ cells are predominantly B cells [45]. IgE+ monocytes were CD23- in both allergic (Fig 3D; median 96.8%, range 96.0–99.8% CD23- out of total IgE+ monocytes) and nonallergic horses (Fig 3D; median 96.5%, range 87.4–98.4% CD23- out of total IgE+ monocytes). Expression of αγ FcεRI mRNA in combination with the lack of FcεRII/CD23 expression suggested that IgE on the surface of monocytes is bound through the trimeric high affinity FcεRI receptor (αγ_2_) and that these cells are IgE-binding monocytes.

### IgE-binding monocyte frequency and changes in CD16 expression during *Cul* exposure

**Fig 4:**
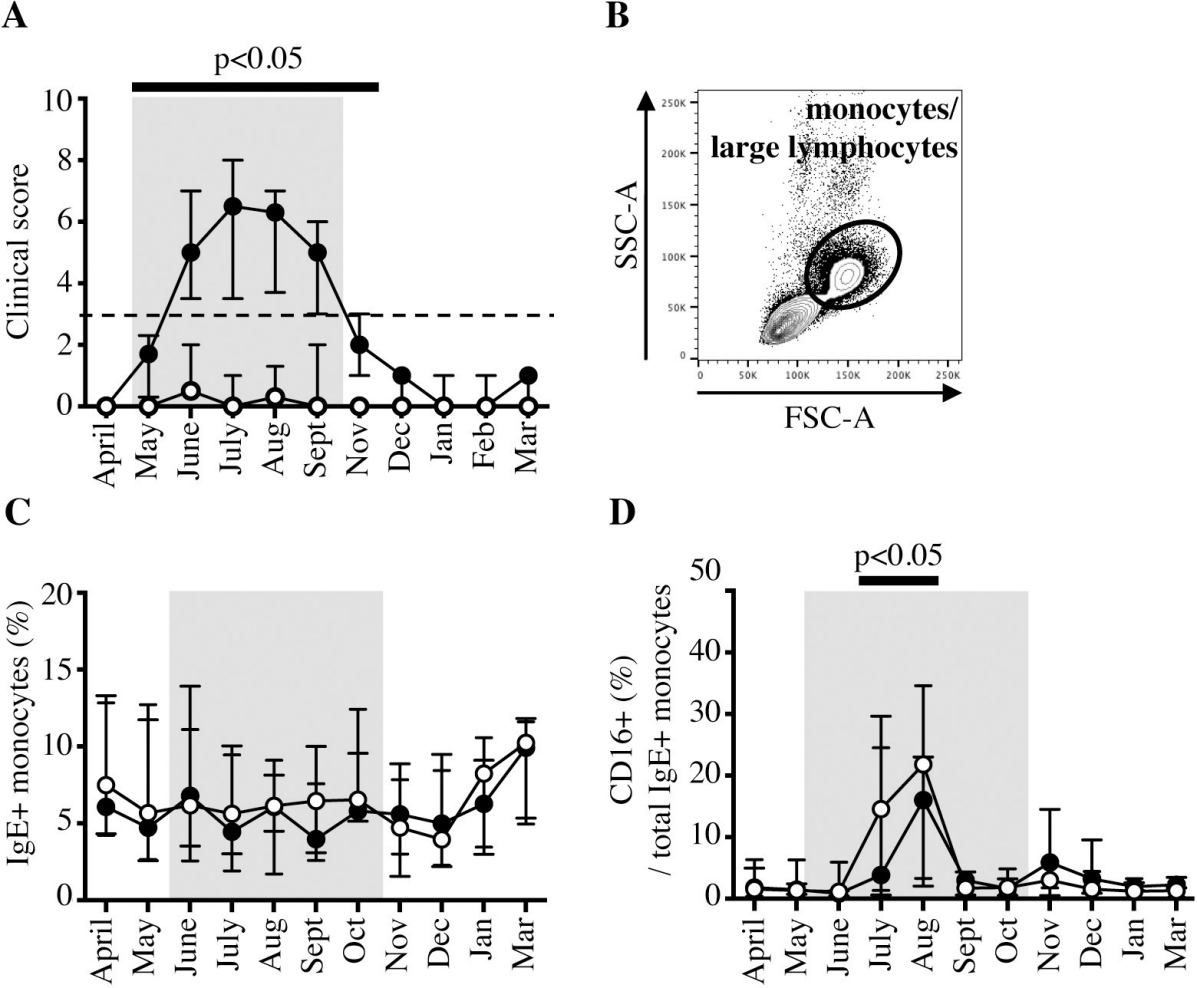
IgE-binding monocytes increase CD16 expression after environmental *Cul* exposure. IgE-binding monocyte (IgE+/CD14^low^/MHCII^high^) surface protein expression was compared between allergic (closed circles, ●, n = 7) and nonallergic (open circles, ○, n = 7) horses living in the same environment with *Cul* exposure from mid-May through September (bolded months, gray shaded box). (A) Clinical allergy scores. The horizontal bar and p-value indicate months with significantly higher clinical allergy scores in the allergic group compared to the nonallergic group. (B) Monocytes and large lymphocytes were gated by forward (FSC) and side scatter (SSC) morphology. (C) Percentages of IgE-binding monocytes out of total peripheral blood monocytes/large lymphocytes. (D) Percentage of CD16+ cells out of total IgE-binding monocytes. The bar and p-value show the months in which CD16 expression was significantly upregulated in both allergic and nonallergic groups compared to CD16 expression in April.

To determine whether IgE-binding monocytes displayed phenotypic differences following changes in *Cul* exposure or between allergic and non-allergic horses, PBMC samples were analyzed monthly by flow cytometry for one year. As expected, clinical signs in allergic horses increased shortly following *Cul* exposure, while nonallergic horses remained clinically healthy (Table 1, Fig 4A). To compare IgE-binding monocytes between allergic and nonallergic groups by flow cytometry, we first gated for monocytes/large lymphocytes by forward (FSC) and side scatter (SSC) characteristics (Fig 4B). IgE-binding monocytes consistently comprised about 6% of the total monocyte/large lymphocyte population (median 6.13%, range 1.56–13.92% out of total monocytes/large lymphocytes). Independent of *Cul* exposure, allergic and nonallergic horses displayed similar percentages of IgE-binding monocytes in their peripheral blood at all timepoints during the year (Fig 4C). However, CD16 expression on IgE-binding monocytes was upregulated following initial *Cul* exposure in most horses and significantly higher in July and August (median 15.3%, range 0.6–34.6%) compared to April (median 1.7%, range 0.4–6.3%) when *Cul* were not present (Fig 4D). Differences in CD16 expression on IgE-binding monocytes between allergic and nonallergic groups were not observed.

### Seasonal dynamics of IgE binding on the surface of IgE-binding monocytes

**Fig 5:**
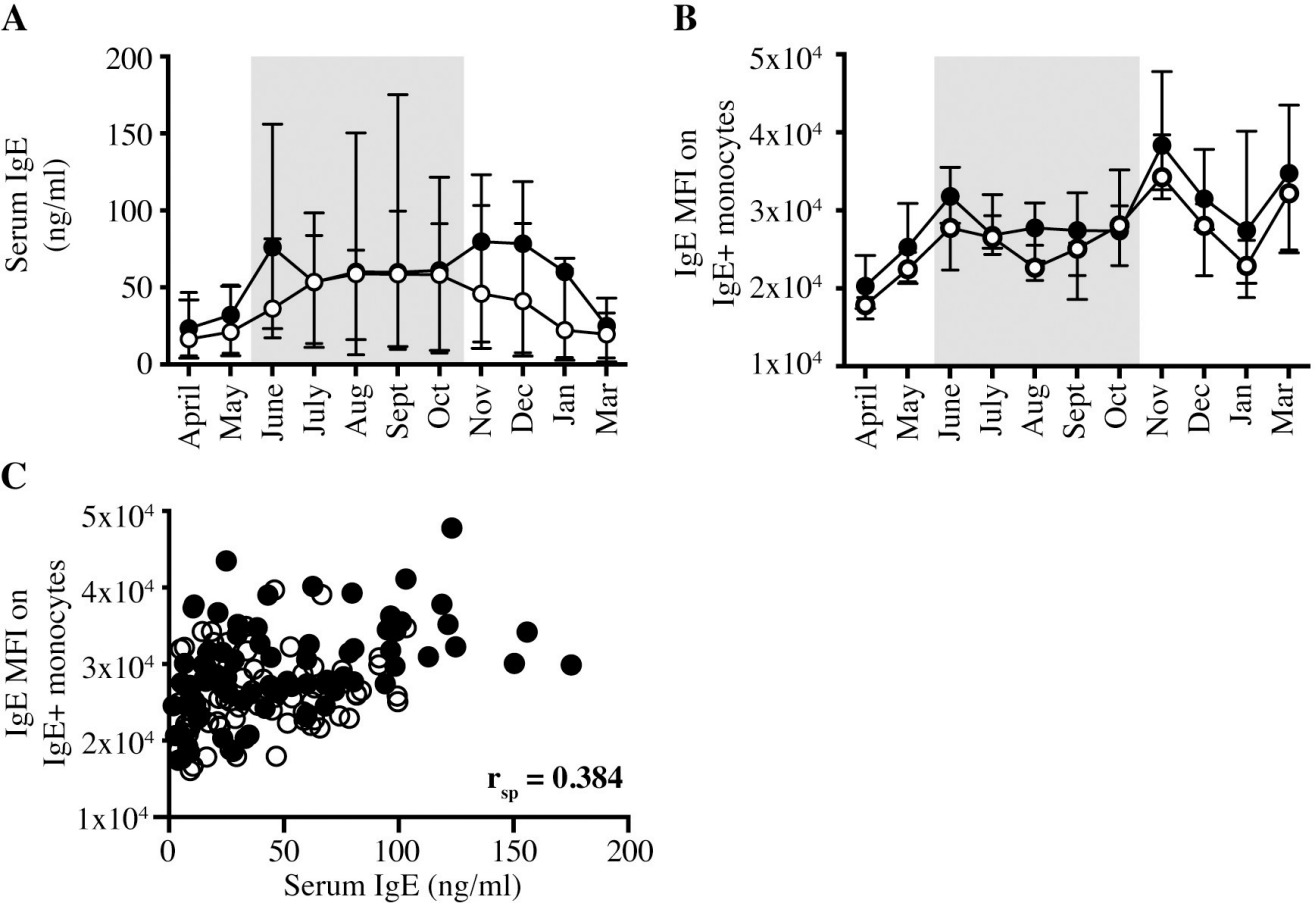
Serum total IgE concentration and monocyte surface IgE density are similar between allergic and nonallergic horses. Total IgE in serum and on monocytes was measured and compared between allergic and nonallergic horses. Graphs compare allergic (closed circles, ●, n = 7) and nonallergic (open circles, ○, n = 7) horses. Months with *Cul* exposure and clinical signs in allergic horses are denoted by bolded months and a gray shaded box. (A) Serum total IgE concentration (ng/ml). (B) IgE Median Fluorescence Intensity (MFI) on IgE-binding monocytes (IgE+/CD14^low^). (C) The correlation between serum total IgE concentration and IgE MFI on IgE-binding monocytes was compared for all horses at all time points tested (April 2018 –March 2019).

Increased serum total IgE concentrations have been correlated with increased FcεRI on the surface of basophils and monocytes in humans [38,50]. We compared total IgE concentrations in serum to IgE densities on the surface of monocytes in both horse groups for one year. All horses, regardless of signs of clinical allergy, exhibited similar concentrations of total IgE in serum at all timepoints throughout the year (Fig 5A). IgE density on the surface of IgE-binding monocytes was quantified as median fluorescence intensity (MFI) by flow cytometry. IgE MFI on IgE-binding monocytes was similar in allergic and nonallergic horses (Fig 5B). When we compared these two parameters in all horses at all seasonal timepoints, the Spearman r correlation coefficient (r_sp_) between serum total IgE and IgE on the surface of monocytes was 0.384. (p < 0.0001; Fig 5C) This suggested only a moderately positive correlation between total IgE concentrations in serum and the amount of IgE bound to the trimeric FcεRI on IgE-binding monocytes in horses.

### IgE-mediated IL-10 secretion by IgE-binding monocytes

**Fig 6:**
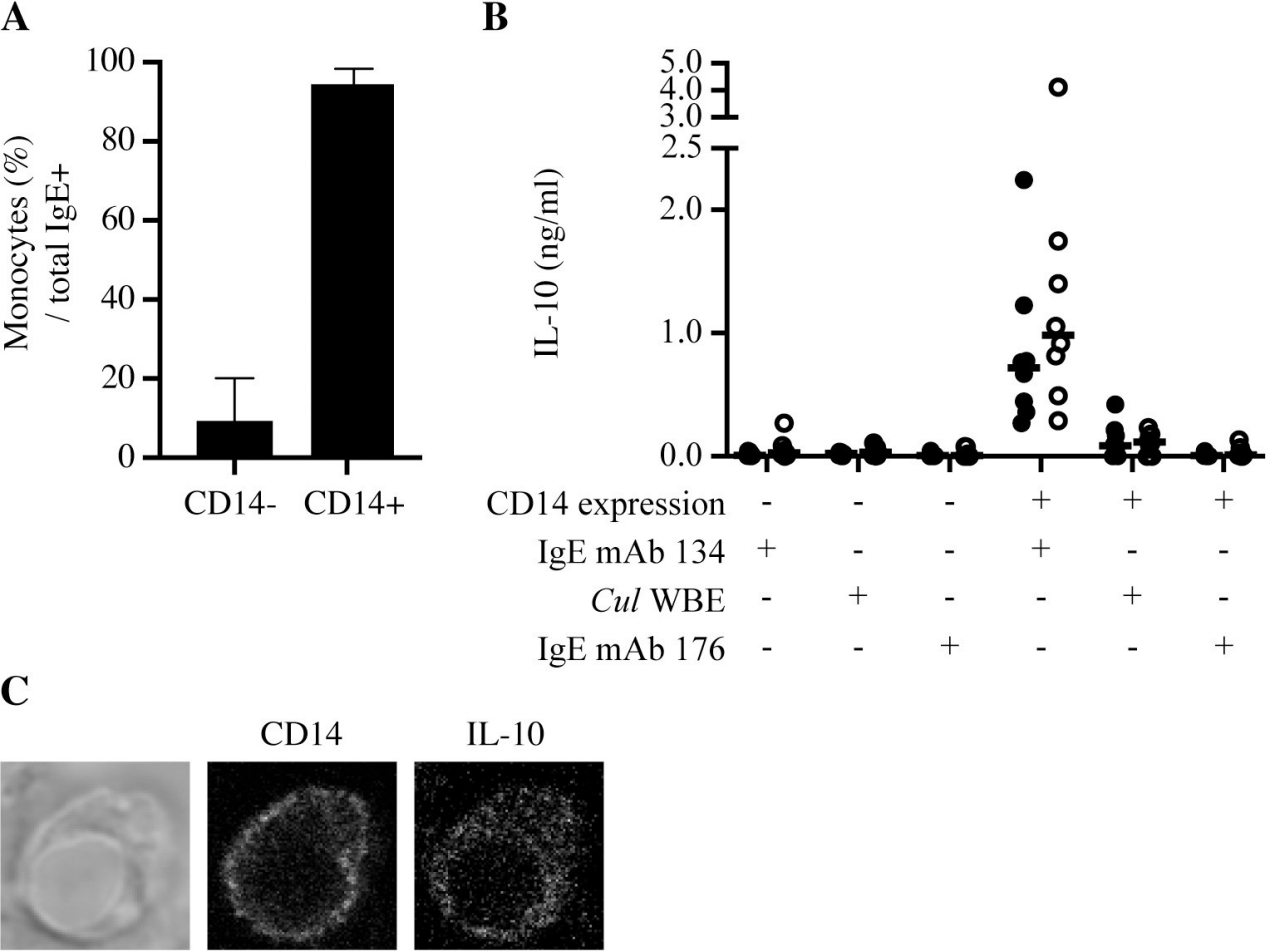
IgE-binding monocytes secrete IL-10 after IgE crosslinking. IgE-binding monocytes were enriched from PBMC by magnetic cell sorting of CD14+ cells. (A) Percentages of IgE-binding monocytes (IgE+/CD14^low^) out of total IgE+ cells in sorted fractions were analyzed by flow cytometry (n = 16) (B) CD14- and CD14+ cells were stimulated with crosslinking mAb (IgE mAb 134), *Cul* WBE, or non-crosslinking mAb (IgE mAb 176) for 48 hours. Secreted IL-10 in the supernatants of cultured cells was analyzed by a cytokine bead-based assay. Individual horse values and medians are graphed for allergic (closed circles, ●, n = 8) and nonallergic (open circles, ○, n = 8) horses. (C) CD14+ cells were stimulated with IgE mAb 134 for 24 hours and Brefeldin A for last 4 hours. Cells were fixed and labeled with anti-CD14 and anti-IL-10 mAbs and imaged by confocal microscopy at 65x with a 16-bit image.

Monocytes were enriched by magnetic cell sorting and stimulated with an anti-IgE monoclonal antibody or *Cul* WBE to determine which cytokines were secreted by IgE-binding monocytes following IgE crosslinking. PBMC were sorted into two fractions: CD14- cells depleted of IgE-binding monocytes, and CD14+ cells containing IgE-binding monocytes (Fig 6A). CD14+ cells stimulated with crosslinking IgE mAb 134 secreted IL-10 (Fig 6B) but not IL-4, IL-17A, or IFN-γ (Table J in S2 Fig). CD14+ cells stimulated with *Cul* WBE also secreted IL-10 but at lower concentrations compared to total IgE crosslinking (Fig 6B). IL-10 concentrations produced by CD14+ cells from allergic and nonallergic horses were similar. CD14- cells stimulated with the same crosslinking IgE mAb 134 or *Cul* WBE did not produce any IL-10. As a negative control, both CD14- and CD14+ cells were stimulated with non-crosslinking IgE mAb 176 and also did not produce any IL-10 (Fig 6B), IL-4, IL-17A, or IFN-γ (Table J in S2 Fig). This confirmed that IgE mAb 176 incubation with live CD14+ monocytes did not induce IgE receptor crosslinking or IgE-mediated cytokine production and can be used as a negative non-crosslinking control.

IL-10 production was also visualized intracellularly by confocal imaging of CD14+ sorted cells stimulated with crosslinking IgE mAb 134 and stained for intracellular IL-10 (Fig 6C). These data collectively supported that IgE crosslinking induces IL-10 secretion, but not other cytokines, by CD14+ IgE-binding monocytes.

## Discussion

IgE-mediated Type I hypersensitivities are initiated by B cell production of allergen-specific IgE, followed by rapid degranulation of inflammatory mediators upon IgE crosslinking of mast cells and basophils [15–18,51–53]. While these cellular mediators remain critically important, it is necessary to broaden the mechanistic understanding about pathogenesis and regulation of allergic diseases. This will improve our ability to identify effective and eventually also causative treatments for IgE-mediated allergies. Here, we focused on the most common IgE-mediated allergic disease in horses, *Culicoides* hypersensitivity, and characterized for the first time the phenotypic and functional properties of IgE-binding monocytes in the horse.

Human IgE-binding monocytes and dendritic cells have been described previously and are capable of activating T cells with processed antigen from internalized allergen/IgE/FcεRI immune complexes [20,28–30,40]. However, how IgE-binding monocytes communicate with and contribute to local or systemic immune reactions and the immune cell microenvironment during an allergic reaction is not yet fully known. Human IgE-binding monocytes were previously identified and characterized in peripheral blood by surface protein expression of CD14+/MHCII+/CD16- and an αγ_2_ variant of FcεRI [19,20,39,50]. FcεRI expression on human IgE-binding monocytes was increased in patients with atopic disease and positively correlated with serum IgE concentration [19,20,38,50]. Additionally, in response to IgE receptor crosslinking, IgE-binding monocytes from atopic individuals secreted IL-10, IL-1β, IL-6, IL-8 and TNF-α [39,41], and exhibited impaired phagocytosis [39]. These data suggested that human IgE-binding monocytes respond to allergen/IgE complexes and behave differently in allergic human patients compared to healthy control individuals. IgE-binding monocytes have not been identified in mice [54].

In this article, we characterized equine IgE-binding monocytes in horses with or without *Cul* hypersensitivity and found overall striking similarities to their human counterparts. Equine IgE-binding monocytes are IgE+/CD14^low^/MHCII^high^/CD16-/CD163-/CD23- cells, express FcεRI α and γ, but not β, subunit mRNA, and represent about 6% of the total peripheral monocytes. However, equine IgE-binding monocytes bound similar densities of IgE on their surface, regardless of clinical signs of allergy or environmental exposure to *Cul*. We expect that IgE MFI directly correlates to surface density of FcεRI because IgE readily sensitizes FcεRI+ cells by binding to the receptor independently from specific allergen/ligand binding at a FcεRIα chain to IgE ratio of 1:1 [23,52]. Our results on IgE densities on equine IgE-binding monocytes are in contrast to human studies which showed that IgE-binding monocytes in humans with atopic disease had increased surface FcεRI expression [19,20,38,50].

It is accepted that human IgE stabilizes FcεRI upon binding and upregulates FcεRI surface expression on basophils and mast cells [52,55,56]. Conflicting studies have shown that human monocytes either do or do not upregulate FcεRI through a similar mechanism [38,57]. With our horse study, all allergic and nonallergic horses had similar serum total IgE concentrations and similar densities of IgE on monocytes. The correlation between serum total IgE and monocyte surface IgE was only moderately positive. These results do not fully support the hypothesis that FcεRI on IgE-binding monocytes is generally upregulated upon IgE binding. Most likely, additional factors such as environmental exposure, parasite burden, individual immune response variations, and possibly others, are also influencing IgE receptor crosslinking dynamics, turn-around times and the regulation of FcεRI expression.

Following phenotypic characterization of IgE-binding monocytes in peripheral blood, we sorted equine monocytes for *in vitro* stimulation to assess IgE-mediated cytokine secretion. Consistent with human studies, equine monocytes produced IL-10 in response to IgE receptor crosslinking and also at low concentrations in response to *Cul* WBE. However, while human studies detected increased IL-10 production in individuals with atopic disease [39,41,58], in our controlled horse model, we demonstrated that IgE-binding monocytes from both allergic and healthy horses secreted IL-10 in response to IgE crosslinking. IL-10 secreted in response to *Cul* WBE was also similar between allergic and healthy horses and at much lower concentrations compared to total IgE crosslinking.

Previous studies [59,60] identified IL-10 as a key down-regulator of IL-4 production by equine T cells but failed to determine the cellular source. These studies hypothesized that IL-10 promoted allergen tolerance in healthy horses. In our current study, we provided evidence that IgE-binding monocytes are the primary producers of IL-10 in response to IgE crosslinking. There was a trend towards increased IL-10 production by IgE-binding monocytes in nonallergic horses. We hypothesize this could contribute to a downstream decrease in IL-4 producing cells, subsequent induction of allergen tolerance, and elimination of clinical allergic disease. However, individual variations in IL-10 production in both allergic and non-allergic horses and the lack of a statistically significant difference in IL-10 secretion between the two groups in our approach do not fully support the importance of monocyte-derived IL-10 as a key regulator of *Cul* hypersensitivity. Most likely, other IL-10 producing cells, such as T regulatory cells, are majorly responsible for the regulation of *Cul* hypersensitivity [46].

The stimulation experiments in this study were conducted during the end of *Cul* exposure (October). We observed that IL-10 production by IgE-binding monocytes varied between seasons with or without *Cul* exposure. Future exploration of IL-10 production throughout changes in allergen exposure may highlight a temporal importance for IL-10 production leading to allergen tolerance, or may increase the importance of looking for more robust indicators of immune regulation in *Cul* hypersensitivity. It is also possible that additional regulatory factors may contribute to IgE-binding monocyte mediated allergen tolerance in nonallergic horses, but not allergic horses.

Our IL-10 production results stand in contrast to human studies, which showed increased IgE-mediated IL-10 production only in humans with atopic disease (mixed populations of patients had asthma, allergic rhinitis and/or atopic dermatitis). IL-10 has canonically been attributed to the development of immune tolerance [61,62]. However, there is some evidence that depending on the local environment, IL-10 may act to promote allergic inflammation in response to IgE stimulation. Specifically, IL-10 has been shown to enhance IgE secretion by isotype switched B cells and Th2 differentiation by CD4+ T cells [58,63]. A C-A polymorphism in the IL-10 promoter region has also been identified in IL-10 producing cells in humans with atopic disease and the presence of this polymorphism was positively correlated with increased serum total IgE concentrations [64,65]. Together, these reports emphasize the need for well-controlled experiments that will sort out exactly how IL-10, and more specifically IgE-binding monocytes, contribute to allergic disease or allergen tolerance in the context of specific allergen stimulation.

While human studies present multiple differences between IgE-binding monocytes in healthy and allergic individuals that could serve as predictive biomarkers of disease, the results in our equine model do not confirm IgE-binding monocytes as biomarkers of *Cul* hypersensitivity. One possible explanation for the differences between prior human studies and our horse study could be due to uncontrolled variables inherent to human cohorts. Similar uncontrolled variables, differences in housing environments, and seasonal variation between sample collection timepoints also applied to previous horse studies on *Cul* hypersensitivity [2,10,12,16–18]. In our study, the use of the equine model allowed the study of a naturally occurring allergic response in a controlled environment (same geographic location, housing conditions, diet, vaccination and deworming schedules) with comparable *Cul* exposure between all horses. Human atopic disease is a highly heterogeneous condition that develops in response to a wide range of inducing allergens [66], while *Cul* hypersensitivity is an allergic reaction against known and characterized allergens [34–36]. As a result of keeping environmental variables similar between horses, and also studying one consistent and well-established allergic disease, we can define similarities and differences in cellular function in the equine model that are due specifically to allergen exposure and lack of tolerance.

We also detected upregulation of CD16 on IgE-binding monocytes in all allergic and nonallergic horses during the summer months. CD16 (FcγRIII) is an IgG receptor that is primarily found on monocytes and large lymphocytes in horses, but some CD16+ granulocytes and lymphocytes are also present [43]. FcεRI and CD16 are both multi-subunit receptors that share the same ɣ chain [67]. The γ chain in these receptors comprises the ITAM (immunoreceptor tyrosine-based activation motif) intracellular signaling portion of the receptor [67] and therefore links the signaling through both IgG/CD16 and IgE/FcεRI. Thus, IgE- and IgG-mediated responses may influence functions of IgE-binding monocytes such as antigen-presentation and/or immune regulation. We hypothesize that IgE-binding monocytes upregulated CD16 in response to environmental changes in the summer, including insects and allergens. However, our study did not determine allergen-specific responses of IgE-binding monocytes, and therefore further work is necessary to identify the causes of increased CD16 on IgE-binding monocytes, and to clarify if CD16 upregulation is allergen-specific. CD16 was upregulated on IgE-binding monocytes in nonallergic horses and also on IgE-/CD14+ monocytes in the summer months (Table K in S2 Fig), suggesting that CD16 may be upregulated through an IgE and allergen-independent mechanism.

The initial characterization of IgE-binding monocytes in the horse, the second species besides humans in which these cells have now been identified, will enable us to further explore mechanistic and regulatory functions of IgE-binding monocytes specifically in response to allergen and IgE stimulation. In the horse model this can be performed with fewer confounding variables than in humans. In order to further characterize the mechanistic roles of equine IgE-binding monocytes during allergic responses, immunologic, genomic and proteomic approaches will provide valuable unbiased insight into the inflammatory or anti-inflammatory bias of IgE-binding monocytes in response to IgE signaling.

## Conclusions

IgE-binding monocytes can be identified and characterized in peripheral blood of horses as IgE+/CD14^low^/MHCII^high^ cells. Overall, IgE-binding monocyte characteristics and functions were similar in both clinically healthy and allergic horses. They bind IgE via an αγ variant of FcεRI lacking the β chain. Monocyte surface IgE was moderately positively correlated to serum total IgE in all horses throughout the year. Following environmental *Cul* exposure, IgE-binding monocytes upregulated CD16 expression. In all horses, IgE-binding monocytes secreted IL-10 upon IgE crosslinking.

## Supporting information

S1 Fig Uncropped Fig 3A gel images of FcεRI α, β, and γ subunit genes. RNA was extracted from snap-frozen cells (PBMC, MACS sorted CD14- cells, MACS+FACS sorted CD14+/IgE- cells, and MACS+FACS sorted IgE-binding monocytes (IgE+/CD14^low^/MHCII^high^/CD16-/CD163-), converted to cDNA, and amplified with gene specific primers for equine FcεRI α, β, and γ mRNA. RNA samples were also amplified as a negative control. Samples were loaded onto a 1% agarose gel, run at 90V/400A and imaged with GelRed. All cells came from the same horse on the same day. PCR reactions and gels were run simultaneously. White boxes denote cropped images included in Fig 3A.
(TIF)

S2 Fig Individual horse values used to calculate medians and ranges for Figs 2–6. Individual percentages, median fluorescent intensities and concentrations for each horse that were used to generate figure graphs are compiled in labeled data tables. (A) Percentage of IgE+ monocytes out of total cells in unsorted, MACS sorted and MACS+FACS sorted samples from 18 different horses in Fig 2D. (B) Percentage of CD23- cells out of total IgE+ monocytes in Fig 3D. (C) Clinical scores of allergic in in Fig 4A. (D) Percentage of IgE+ monocytes out of total monocytes in Fig 4C. (E) Percentage of CD16+ cells out of total IgE+ monocytes in Fig 4D. (F) Serum total IgE (ng/ml) measured by bead-based assay in Fig 5A. (G) IgE median fluorescent intensity (MFI) of IgE mAb 176 (Alexa Fluor 488) on IgE+ monocytes in Fig 5B. (H) Combined serum total IgE and IgE MFI on IgE+ monocytes in Fig 5C. (I) Percentage of monocytes out of total IgE+ cells in Fig 6A. (J) Secreted concentration of IL-10 (pg/ml), IL-4 (pg/ml), IFN𝛾 (MFI) and IL-17A (MFI) as measured by bead-based assay in Fig 6B. (K) Percentage of CD16+ cells out of total IgE- CD14+ monocytes. B-H,K show allergic (n = 7) and nonallergic (n = 7) horses, J shows allergic (n = 8) and nonallergic (n = 8) horses in October 2019. C-H,K show data points collected from April 2018-March 2019.
(XLSX)

S3 Fig Uncropped Fig 6C confocal images of monocytes incubated with IgE mAb 134. CD14+ MACS sorted cells were incubated in MatTek coverslip wells in the presence of IgE mAb 134 for 24 hours at 37ºC. Cells were fixed and incubated with fluorescently coupled mAbs against CD14 and IL-10. 16-bit images were taken at 65x magnification under (A) brightfield, (B) 488 nm laser excitation of CD14 mAb staining, and (C) 633 nm excitation of IL-10 mAb staining. White boxes denote cropped images included in Fig 6C.
(TIF)
