# Supplementary figures and images for: Phenotype and function of IgE-binding monocytes in equine Culicoides hypersensitivity
Source: PLoS One. 2020 May 22;15(5):e0233537. doi: 10.1371/journal.pone.0233537 (PMC7244122; doi:10.1371/journal.pone.0233537)

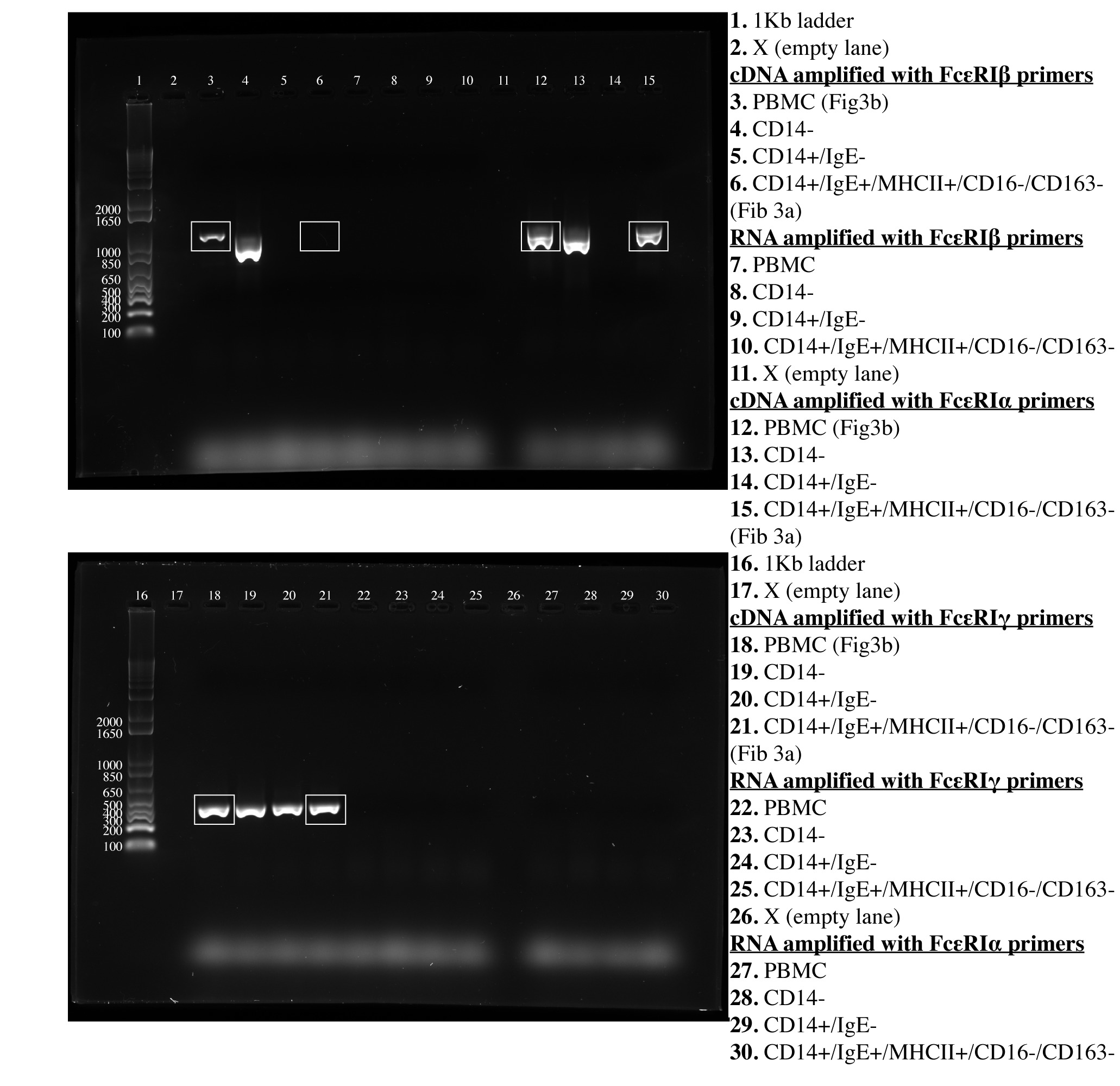

Supplement: S1 Fig — RNA was extracted from snap-frozen cells (PBMC, MACS sorted CD14- cells, MACS+FACS sorted CD14+/IgE- cells, and MACS+FACS sorted IgE-binding monocytes (IgE+/CD14low/MHCIIhigh/CD16-/CD163-), converted to cDNA, and amplified with gene specific primers for equine FcεRI α, β, and γ mRNA. RNA samples were also amplified as a negative control. Samples were loaded onto a 1% agarose gel, run at 90V/400A and imaged with GelRed. All cells came from the same horse on the same day. PCR reactions and gels were run simultaneously. White boxes denote cropped images included in Fig 3A. (TIF) [file pone.0233537.s001.tif]

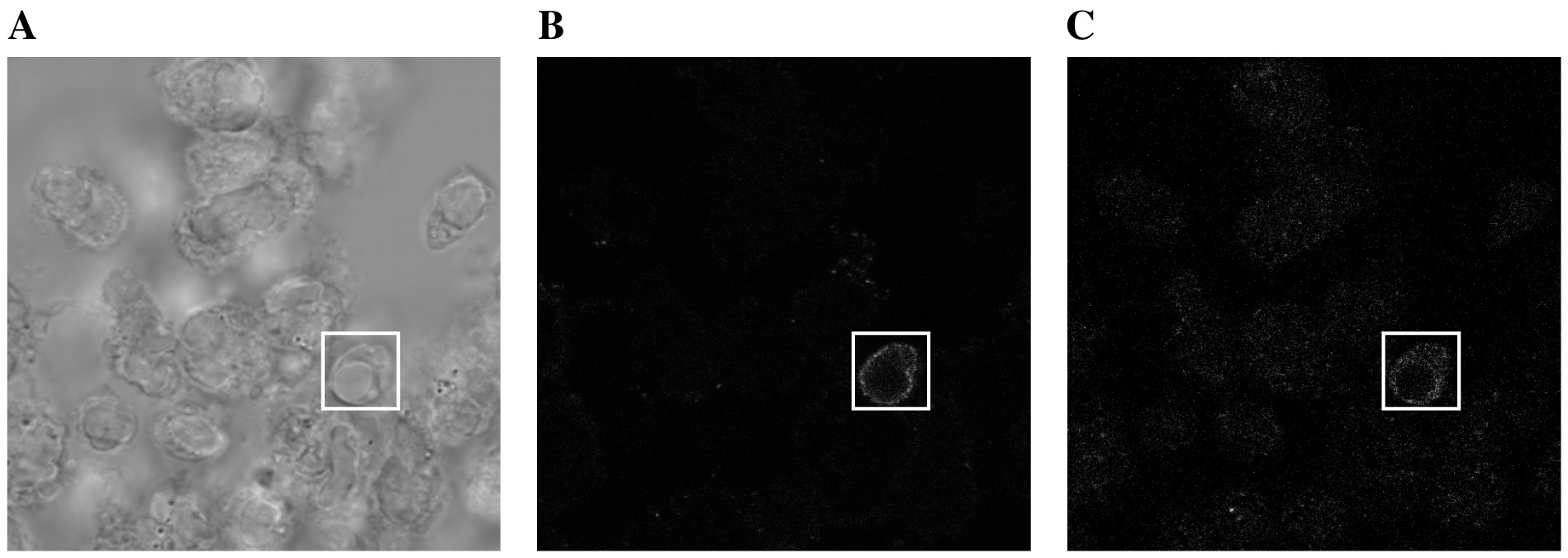

Supplement: S3 Fig — CD14+ MACS sorted cells were incubated in MatTek coverslip wells in the presence of IgE mAb 134 for 24 hours at 37ºC. Cells were fixed and incubated with fluorescently coupled mAbs against CD14 and IL-10. 16-bit images were taken at 65x magnification under (A) brightfield, (B) 488 nm laser excitation of CD14 mAb staining, and (C) 633 nm excitation of IL-10 mAb staining. White boxes denote cropped images included in Fig 6C. (TIF) [file pone.0233537.s003.tif]
